# Evaluation of the Implementation of Integrated Primary Care for Patients with Type 2 Diabetes and Hypertension in Belgium, Cambodia, and Slovenia

**DOI:** 10.5334/ijic.7664

**Published:** 2024-06-28

**Authors:** Nataša Stojnić, Monika Martens, Edwin Wouters, Savina Chham, Josefien van Olmen, Katrien Danhieux, Nina Ružić Gorenjec, Ir Por, Antonija Poplas-Susič, Zalika Klemenc-Ketiš

**Affiliations:** 1 Community Health Centre Ljubljana, Primary Healthcare Research and Development Institute, Ljubljana, Slovenia; 2 Department of Public Health, Institute of Tropical Medicine, Antwerp, Belgium; 3 Department of Family Medicine and Population Health (FAMPOP), Faculty of Medicine and Health Sciences, University of Antwerp, Belgium; 4 Centre for Population, Family & Health, Department of Social Sciences, University of Antwerp, Antwerp, Belgium; 5 Centre for Health Systems Research & Development, University of the Free State, Bloemfontein, South Africa; 6 National Institute of Public Health, Cambodia; 7 Department of Social Sciences, University of Antwerp, Belgium; 8 Department of Family Medicine, Faculty of Medicine, University of Ljubljana, Ljubljana, Slovenia; 9 Department of Family Medicine, Faculty of Medicine, University of Maribor, Maribor, Slovenia

**Keywords:** integrated care package, primary care, cross country comparison, diabetes, hypertension

## Abstract

**Introduction:**

Integrated care of chronic patients improves quality of their management, but there is scarce evidence of its implementation in different healthcare settings. With this article, we wanted to determine the level of integrated care implementation in the management of T2D (diabetes) and HT (hypertension) in three different settings: Belgium, Slovenia, and Cambodia.

**Methods:**

This was an observational study with integrated approach. It was conducted in primary health care organisations in three countries. In each primary health care organisation, we aimed to include primary care workers that worked with Type 2 Diabetes (T2D) and hypertension (HT) patients. Data was collected with the Integrated Care Package (ICP) grid (consisting of six elements: identification, treatment, health education, self-management, caregiver collaboration, and care organisation).

**Results:**

ICP is almost completely implemented without major differences within Slovenia. There is a considerable variability across practice types in Belgium. Implementation is constrained by health system resources in Cambodia. Some elements, especially identification, are better implemented then others, across health systems.

**Conclusion:**

Countries can enhance integrated care for chronic diseases by implementing central policies, standardized protocols, and local adaptation, addressing resource constraints, promoting systematic screening and health education, and providing training for healthcare workers, tailored to community needs, to improve patient outcomes and healthcare delivery.

## Introduction

Non-communicable diseases (NCDs) are on the rise across the globe [1]. This is also reflected in its associated mortality, as 74% of all deaths globally are due to NCDs. It is projected that global deaths from NCDs will continue to rise over the next several decades, primarily due to aging populations [2]. The global prevalence rates for type 2 diabetes (T2D) (9.3%) and hypertension (HT) (31.1%) reflect the dominance of these two conditions in the global burden of disease [3]. Their prevalence is high in both low- and middle/high income countries [4], especially among vulnerable populations.

However, the health system response to this public health problem is challenging as care for people with NCDs is organisationally complex: it requires continuous and concerted action by different actors. In contrast, a fragmented care management model that is not fully integrated in the system is still dominant in many settings, rendering the success of NCD care often limited [5]. As a response, there has been a shift towards more integrated care for NCDs in the past years in the context of management of chronic patients at primary health care. Integrated care can be described as a coherent and coordinated set of services planned, managed and offered to individual service users by a number of organisations and a range of cooperating professionals and informal carers [6].

We specified an integrated care package (ICP) for T2D and HT with six components: (a) identification of people with disease and subsequent (b) treatment in primary care, (c) health education, and (d) self-management support by patients and caregivers, (e) collaboration between caregivers, and f) coordinated organisation of care [7]. We applied this framework to assess the implementation of ICP for T2D and HT. Integrated care for T2D and HT can provide a solution to several long-standing problems in the health care system, such as the lack of continuity of care, the fragmentation of medical care/treatment processes and the quality of patient education [8]. In general, there is evidence that integrated care improves the quality of care and its outcomes [7,8,9], but there are still significant knowledge gaps relating to implementation and scale-up, such as the feasibility and depth of implementation of ICP elements in different health systems and for different diseases, and which barriers and facilitators interfere with scale up in a particular context [6].

In Belgium the healthcare system is decentralized and privatized, but regulated and based on the concepts of independent medical practice, freedom of choice of physician and care facility, and primarily on fee-for-service payment [10]. While several initiatives have been initiated in Belgium over the last decade to scale up integrated care, the stakeholders still consider the current level of implementation of integrated care to be low. The main barriers to scale-up of integrated care are the payment model for services, limited data exchange and division of responsibilities among various levels of government [11]. COVID-19 has forced healthcare professionals in Belgium and elsewhere to adopt interdisciplinary approaches and new processes. In addition, the importance of respectful and person-centred care, especially for older adults, is increasingly recognised [12].

In Slovenia primary health care is managed by municipalities. It offers comprehensive and patient-centred health care by multi-disciplinary teams comprising various medical professionals like family physician, pediatricians, primary gynaecologists and dentists. Services include emergency care, dentistry, diagnostics, therapy, mental health support, nursing, and health education. This system emphasizes community-oriented care and provides a wide range of preventive, diagnostic, treatment, and wellness services [13]. Overall satisfaction with healthcare is good. There’s a growing need for preventive care and improved care coordination, especially for those with chronic conditions. Despite high co-payments for many services under universal compulsory health insurance, most people are covered by voluntary health insurance, offsetting these costs for 95% of the population [14].

Cambodia’s healthcare system encompasses both public and private sectors [15]. They face challenges that include financial constraints, a shortage of healthcare professionals, a shortage of medicines, poor management of health information, problems in the provision of healthcare services and poor governance, which could hamper efforts to scale up services for diseases such as T2D and HT [15]. The Ministry of Health (MoH) acknowledges the significance of the Integrated Chronic Care Program (ICP) and is committed to its implementation through several interventions. These include: (1) Establishing Non-Communicable Disease (NCD) clinics at referral hospitals (RHs), (2) Introducing the World Health Organization Package of Essential Non-Communicable Disease Interventions (WHO PEN) program in health centers (HCs), and (3) Expanding and integrating the community-based MoPoTsyo’s Peer Educator Network, where train peer educators provide community-based care and support for people with T2D and HT [16,17,18]. Currently, there are three main approaches to delivering ICP within an operational district (OD): a) ODs with a hospital-based diabetes clinic only; b) ODs with a diabetes clinic and health centres that perform PEN-identified tasks; c) ODs with community-based patient support collaborating with the district hospital. In some ODs a, b and c are combined [7].

In these three countries, we aimed to 1) assess the implementation of the ICP for two priority diseases being T2D and HT and 2) identify the contextual and health system factors facilitating or impeding the implementation of the ICP to provide insights on how to facilitate scale-up of integrated care, considering the health system and societal context.

## Methods

### Design and study population

This was an observational study with integrated approach, where qualitative information was transformed into quantitative data for analysis. The research questions were addressed using an ICP grid questionnaire that enables assessment of multiple resources to select the final answer. It was conducted in three countries with different types of health systems and societal context: a centralized health system in high-income country in Slovenia, a decentralized system in high-income country in Belgium, and a developing health system in lower middle-income country in Cambodia. This study is part of a larger study, named ‘Scaling up an integrated diabetes and hypertension care package for vulnerable people at risk in Cambodia, Slovenia and Belgium’ (SCUBY) [7,9].

The prime target population for the study were health care organisations essential in the primary care provision of T2D/HT care in each country. These were family medicine teams in Slovenia, primary care practices in Belgium and a combination of health centres and hospitals in Cambodia. To capture the variation in organisation within each country, sampling of health organisations was done purposively, the process for each country is described below.

In Slovenia, the research was conducted in three regions, representing different development contexts: 1) in capital Ljubljana catering to around 300,000 residents, representing an urban profile; 2) Ravne na Koroškem, a rural north Slovenian area, covering around 11,000 residents; and 3) Lendava, a rural northeast Slovenian area, and covering 10,000 residents. We selected 8 health centres (HCs) from Ljubljana (due to its size), one from Ravne na Koroškem and one from Lendava. For each participating HC we included one extended family medicine team involved in the management of patients with diabetes and/or hypertension participated. Each team involved a family physician, a practice nurse, a registered nurse, a registered nurse working in a health education centre and a community nurse [19,20]. To sum up, in Slovenia 10 family medicine teams participated, 8 from urban and 2 from rural regions.

In Belgium, primary care practices are independent and differ in many ways, including size and administrative support. The majority of practices consist mainly of general practitioners working as individual or group practice, while a minority also includes other healthcare professions such as dieticians or nurses. Primary care centres have different organisational models, including monodisciplinary general practices, multidisciplinary health centres with support from health educators or dieticians and multidisciplinary health centres that operate on a capitation basis, where patients register and the centre receives a fixed fee. In Belgium, 66 primary care practices of three types (multidisciplinary fee-for service, monodisciplinary fee-for service and multidisciplinary capitation-based practices) spread across three regions participated, including two urban areas (Antwerp and Ghent) and one rural (the Campine).

In Cambodia, the study was conducted at five purposively selected operational districts (ODs): OD Daunkeo, OD Kong Pisei, OD Sort Nikum, OD Samrong and OD Pearaing. The selection of the five Cambodian ODs was with a specific focus on three different types of care organisation models described in the introduction and in (a) hospital-based care; (b) health center-based care and (c) community-based care. From each OD three health centres (HCs) and one referral hospital (RH) participated, thereby 20 health facilities (5 RHs + 15 HCs) in total.

SCUBY country team researchers visited each selected health care organisation to assess the ICP implementation and to interview health care workers. Health care worker selection was based upon convenient sampling, aiming to interview any health care worker involved in the care process who was present at the moment of the visit. The respondents vary as the composition of the primary health care teams varies from country to country. All participants gave oral informed consent to participate in the study.

### Data collection

Data collection took place in 2019 to 2022 (it was extended due to COVID-19 pandemic).

To an ICP implementation assessment grid was used for the collection of the data, which served as a researcher-completed survey or checklist. It was developed among the members of the project team from all three participating countries as follows: First, a review of the appropriate tools already available on the topic was performed. Subsequently, the project team identified two validated tools: the Assessment of Chronic Illness Care form (ACIC) [21] and the Assessment of Innovative Care for Chronic Disease framework tool (ICCC) [22]. Both are being used in high and low/middle income settings to assess the degree of implementation of integrated chronic care. However, as they are not disease specific, the project team tailored them to T2D and HT by adding specific questions about these diseases. Then, the tool was translated from English to the national languages and accompanied with a context-specific interview guide that contextualised the terminology in the tool. In each country, it was tested for understandability and feasibility among the users (members of the family practice team) and final changes were made. The resulting ICP grid tool consists of 6 elements: Identification (8 items), Treatment (15 items), Health Education (8 items), Self-Management support (13 items), Structured Collaboration (10 items related to care coordination, relationship with community, cooperation between different health care levels), and Care Organisation (6 items including quality improvement activities, feedback, appointment services). Items of the ICP grid were answered on a Likert scale from 0 to 5 (0 – no implementation of the ICP, 1 – little implementation of the ICP, 2–3 – moderate implementation of the ICP, 4 – almost complete implementation of the ICP, 5 – full implementation of the ICP). For each of the items on the ICP Grid, we defined what each individual score meant/described per country team (contextually). The tool was adapted to the country context. In Cambodia, the self-management element comprised only 11 items. In Belgium, a standard score was determined for 8 items which applied to all practices.

The ICP Grid served as the framework for data collection, facilitating the evaluation of various resources to determine the results. Our approach encompassed multiple data sources: a thorough examination of current health policies and available protocols; on-site observations of facility infrastructure, workflow organization, patient dynamics, and interactions between patients and healthcare personnel; information from the team members specializing in chronic patient care; and scrutiny of facility documentation, including management records, patient registries, and occasional random file checks.

Upon gathering data from these diverse sources, two researchers independently completed the ICP Grid. Subsequently, they compared their assessments and reached a consensus, generating a unified score for each item within the Grid. In instances where consensus couldn’t be reached due to divergent scores, a pre-designated supervisor, possessing expertise in family medicine and qualitative research methodology, intervened to facilitate agreement.

### Data analysis

To obtain valid scores – reflecting the same ICP dimensions in the three different settings which each have varying health care organisation models – we constructed a procedure adapted to each context (Table 1). In Slovenia and in Belgium, the unit of analysis were a Family medicine team and a Primary Care Practice respectively and in Cambodia, the unit of analysis was a health centre or referral hospital. In all countries, each unit received a score for each ICP element calculated as a mean value of the corresponding items, and an overarching ICP score which was calculated as a mean of scores from the six Elements.

**Table 1:** Overview of the contextualised data collection and analysis.


	SLOVENIA	BELGIUM	CAMBODIA

Unit of sampling	Family medicine team	Primary Care Practice	Health centre (HC) or referral hospital (RH)

Units sampled, purposive selection criteria	10 units Regions: - 1 urban regions (n = 8) - 2 rural regions (n = 2)	66 units Organisational types: -monodisciplinary fee-for-service (n = 30) -multidisciplinary fee-for-service (n = 19) -Multidisciplinary, capitation (n = 17)	20 units, 15 HC and 5 RF Organisational types: -hospital-based care -health center-based care -community-based care Units in each operational district (OD): 3 HCs and 1 RH

Respondents in health care organisations	family physicians, practice nurses, registered nurses (working in health centre/health education centre /community)	family physicians, primary care nurses, dieticians	physicians, nurses, mid-wives, community health workers

ICP assessment grid analysis	for every unit, one score for each ICP element, calculated as mean of the corresponding items		

Summative scores per region or practice type (Belgium)	means of scores of the selected units		Score for OD for T2D or HT = (mean (3 HC) + RH)/2 Total score for OD = (score for T2D + score for HT)/2

Summative scores per country	means of scores of the regions	NA	NA


In Slovenia, the scores for regions were calculated as means of scores of the selected units, and scores for the whole country were calculated as means of scores for regions (as it was sensible to give urban and rural setting an equal weight in the final score). This analysis was done separately for T2D and HT (where only the questions that correspond to each disease were considered), and finally according to all the questions of the ICP grid.

In Belgium, it was decided to stratify per practice types and report results for both diseases together due to the nature of health system. The scores for every practice type were a mean of all practices of this practice type. The summative score per country was not given due to diversity of health organisation throughout the country.

In Cambodia, the summative score was given per OD as primary health care is provided by a collaboration of organizations within each district. The score for each OD was a weighted mean of OD’s three HCs and one RH, where each HC got weight 0.17 and RH the weight 0.5 (see Table 1 for formula). Analysis was done also separately for T2D and HT. Due to the differences in health system across country it was decided to refrain from assigning a summative score to whole country.

As thoroughly described above, we had to take different units per country and then calculate and report results differently, all due to different healthcare systems. The calculations are not intended as a direct comparison between countries, but to detect differences according to country’s health system and organisation of care.

### Ethics

The individual countries obtained ethics approval from the representative committees. In Slovenia, the National Ethics Committee approved the study (No. 0120-219/2019/4). In Belgium, the Ethical Committee University of the University Hospital Antwerp approved the study (ref. B300201940005 and B300201941020). In Cambodia, the research protocol was approved by the National Ethics Committee for Health Research (No 115/NECHR of 29^th^ April 2019).

## Results

### Assessment of the implementation of the ICP in Slovenia

In Slovenia, the scores of the ICP were quite similar for T2D and HT with small variability between different family practise teams. Overall, the scores show good implementation, with highest scores for Identification, Treatment, and Health Education, lower for Structured Collaboration and Organisation of Care and lowest for Self-management Support, which was slightly better implemented for T2D than HT (Table 2).

**Table 2:** Implementation of integrated care for patients with T2D and/or HT in Slovenia.


	E1 – IDENTIFICATION	E2 – TREATMENT	E3 – HEALTH EDUCATION	E4 – SELF-MANAGEMENT SUPPORT	E5 – STRUCTURED COLLABORATION	E6 – ORGANIZATION OF CARE	TOTAL SCORE

T2D (range†)	4.9 (4.8–5.0)	3.9 (3.5–4.3)	4.2 (3.8–5.0)	2.9 (2.4–3.6)	3.1 (2.4–3.6)		3.8 (3.6–4.0)

HT (range†)	4.9 (4.8–5.0)	4.1 (3.9–4.3)	4.2 (3.3–5.0)	2.5 (2.0–3.3)	3.1 (2.4–3.6)		3.8 (3.5–4.0)

Total (range†)	4.9 (4.8–5.0)	4.0 (3.7–4.1)	4.2 (3.5–5.0)	2.6 (2.2–3.1)	3.1 (2.4–3.6)	3.6 (2.8–4.2)	3.7 (3.6–3.9)


† Range according to all family medicine teams (from urban and rural regions).

In the interviews, respondents noted the existing protocols for Identification and Treatment. The 2011 nation-wide roll-out of a screening project, with systematic screening for all people above 30, is mentioned as a facilitator factor for the good implementation. Physicians mentioned that pop-up tools in their electronic records could support them in decision-making. A strong facilitator for the implementation of Health Education was the reorganisation of the work in family medicine teams. Namely, the health education task was assigned to the nurse practitioner in 2011. They send structured invitations for group education, although they report that barriers exist for people in vulnerable situations to attend. They express that adapted education materials and different formats such as videos could be facilitators for wider implementation. The relatively low levels of implementation of Self-management Support were linked to a lack of structured, specific, and harmonized guidance across the country, although there was some variability in the score indicating that some family health teams manage to implement this well. Respondents also mentioned that engaging informal care givers and family members could facilitate implementation, especially for vulnerable people such as frail elderly or those with comorbidities. The implementation of element Organisation of Care was reported to be hindered by poor implementation of continuous quality improvement, such as the lack of feedback to practices about their quality assessment results.

### Assessment of the implementation of the ICP in Belgium

In Belgium, the implementation varied across the different practice types with higher scores for primary care practices with a capitation payment and lowest for monodisciplinary practices, especially for Health Education, Self-management Support, Structured Collaboration, and Organisation of care (Table 3). Scores represent both diabetes, T2D and HT, together (see Methods).

**Table 3:** Implementation of integrated care for patients with T2D and/or HT in Belgium.


	E1 – IDENTIFICATION	E2 – TREATMENT	E3 – HEALTH EDUCATION	E4 – SELF-MANAGEMENT SUPPORT	E5 – STRUCTURED COLLABORATION	E6 – ORGANIZATION OF CARE	TOTAL SCORE

Monodisciplinary (mean and range)	4.3 (3.3–4.8)	3.6 (3.0–5.0)	0.0 (0.0–0.0)	1.8 (0.8–2.8)	2.2 (1.0–3.4)	1.3 (0.7–3.5)	2.3 (1.6–2.9)

Multidisciplinary fee-for-service(mean and range)	4.3 (4.0–5.0)	3.7 (3.1–4.3)	1.5 (0.0–3.5)	2.3 (1.1–3.3)	2.4 (1.2–4.0)	2.3 (0.7–4.3)	2.8 (2.2–3.3)

Multidisciplinary capitation (mean and range)	4.4 (4.0–5.0)	3.9 (3.1–4.3)	2.5 (0–3.50)	2.6 (1.1–3.3)	3.4 (1.2–4.0)	3.5 (2.3–4.3)	3.3 (2.8–3.9)


Respondents recognized that the addition of a nurse to the practice facilitates implementation of the ICP, but they mentioned major barriers to doing so are lack of regulations for financing. Many monodisciplinary practices also collaborate with other professionals, but doing so under one roof smoothens this process. The scores for Identification were quite similar because they depended for a great part on the (wide) availability of material. Respondents mentioned that guidelines facilitate the implementation of Treatment, but that adaptation to the specific practice context and decision support such as pop-ups facilitates adaptation. Belgium has a national care trajectory that includes structured education for people with T2D. Health Education and Self-management Support were better implemented in the multidisciplinary practices where nurses took up this task, in a structured way and based upon a protocol. Respondents explained the relative low score on element Organisation of Care by the lack of regulations and incentives to improve quality of care.

### Assessment of the implementation of the ICP in Cambodia

In Cambodia, the ICP grid scores are given per OD to outline the performance of the different health interventions, i.e. the different care organisation models (Table 4). The total ICP score was highest in OD Daunkeo, followed by OD KongPisei, OD Pearaing, OD Sort Nikum and OD Samrong. Most of the total ICP scores were moderate, while the ICP score was slightly higher for HT compared to T2D for all ICP elements across the five ODs. OD Daunkeo scored the highest in Identification, Treatment, Health education, and Self-management, while OD Kong Pisei scores the highest in Structured collaboration and Organization of care. For the ODs in which most interventions were present (hospital, health center based and community-based interventions), ICP implementation was overall better. When looking across ICP elements, Identification scored higher than the other five ICP elements, Structured Collaboration lowest.

**Table 4:** Implementation of integrated care for patients with T2D and/or HT in Cambodia.


OD (PROVINCE)	ORGANISATIONAL TYPE	DISEASE	E1 – IDENTIFICATION	E2 – TREATMENT (WITH RH AS TREATMENT REFERRAL)	E3 – HEALTH EDUCATION	E4 – SELF-MANAGEMENT	E5 – STRUCTURED COLLABORATION	E6 – ORGANIZATION OF CARE	TOTAL

OD Daunkeo (Takeo Province)	(a), (b), (c)	T2D	2.9	2.2	2.0	2.0	1.8	2.3	2.4

		HT	4.4	2.6	2.0	2.2	1.8		

		Total	3.7	2.4	2.0	2.1	1.8		

OD Kong Pisei (Kampong Speu Province)	(c)	T2D	1.6	1.6	0.8	1.7	2.3	2.8	2.1

		HT	4.3	2.1	1.1	2.2	2.4		

		Total	3.0	1.9	1.0	1.9	2.3		

OD Sort Nikum (Siem Reap Province)	(a), (b)	T2D	2.5	1.8	1.5	1.5	1.2	2.2	2.0

		HT	4.1	2.4	1.4	1.9	1.3		

		Total	3.3	2.1	1.4	1.7	1.3		

OD Pearaing (Prey Veng Province)	(a), (b)	T2D	2.4	2.1	1.3	1.5	1.3	2.0	2.0

		HT	4.1	2.4	1.6	2.0	1.4		

		Total	3.3	2.3	1.4	1.8	1.3		

OD Samrong (Oddormeanchey Province)	(a)	T2D	2.0	1.9	1.0	1.5	1.1	2.3	1.9

		HT	4.0	2.5	1.6	1.9	1.3		

		Total	3.0	2.2	1.3	1.7	1.2		


(a) Hospital-based care. (b) Health center-based care. (c) Community-based care.

Respondents in Cambodia mentioned as key barriers to ICP implementation the limited competencies of health professionals and limited availability of medications. This was clearly seen in the scores for Treatment. The shortage of staff and the absence of qualified staff and laboratories at health center level affect the capacity to provide treatment at the operational levels. However, in ODs for which strong leadership was reported, implementation was observed to be better. There is also decision-space to use locally raised revenue from user fees, for instance to purchase medicines addressing shortages. Also, the introduction of a national policy on health care improvement was seen as conducive to ICP implementation.

## Discussion

The results of this study illustrate how the implementation of an ICP for priority diseases in three countries with different health systems differs and how the contextual and health system factors influence this. Our findings show that ICP elements Identification, Treatment, and Health Education are almost completely implemented within Slovenia, while Self-management Support stands out as needing the most substantial improvement. There is a considerable variability across practice types in Belgium, and implementation is constrained by health system resources in Cambodia. The qualitative findings provide explanations from the side of implementers on the ground. They point to the following levers for implementation and scale-up of the ICP: central policies and health services support for nation-wide roll-out, guidelines and practical tools for day-to-day use at operational level, and decision-space to locally adapt and steer resources.

Central policies, on screening and treatment, have proven to support uniform implementation in Slovenia. Family medicine practices are managing the chronic patients with a standardised approach, through the use of protocols [20]. Namely, each family medicine practice in Slovenia, regardless of its status and region, uses such standardised protocols and has a standardised team composition for the management of T2D and HT and guidelines on collaboration within the care team and between different providers e.g., health education centres in region, municipalities, clinical specialists on the secondary/tertiary care levels, social workers, and patient associations. In the pluriform health system of Belgium, family physicians have a lot of freedom to decide on practice organisation and collaboration [23]. In Cambodia, the insufficient financial support to health services leads to shortages in competent staff and medication which severely impede ICP implementation nationwide. In all three countries, the relatively new policies on quality improvement are mentioned as important enablers for better implementation of the ICP.

Notwithstanding the national conditions within each health system, the results also illustrate the need for local adaptation. If health care organisations integrate the treatment guidelines into their daily practice and develop support tools such as pop-ups, health care workers can more easily adopt this. Leaders at the local level can support health care organisations by showing commitment and freeing local resources, which increases material possibilities and motivation, as seen in Cambodia.

The results of the study also show how some elements are better implemented than others, across health systems. In all three countries, the Identification was the element best implemented within the care organisations providing primary care. The qualitative results touch upon the difference between systematic (Slovenia) and opportunistic (Belgium) screening and between community-based (some ODs in Cambodia) and facility-based (most other places). Selective screening creates a gap between the well-informed and the less informed and more vulnerable patients, which leads to global recommendations for systematic screening at places easily accessible and to raising awareness.

The presence of guidelines and protocols were important for the implementation of identification, treatment, and education. The interviews further point to the need to support the health workers in implementation through training, providing them with adequate resources and equipment and practical aids. These could not only facilitate implementation of the ICP but contribute to increased job satisfaction.

For the element health education and self-management support, the role of nurses and community health workers appeared pivotal. Our qualitative findings, also confirmed by other studies suggest that nurses work in a very structured way, and that patients perceive to talk more easily with nurses about self-management. Since self-management is an essential part of chronic care, implementation of self-management of chronic patients is a core priority for scale-up [24]. It is necessary to implement different methods to support patients for self-care. These could be peer support, health literacy, telemedicine, and others [25]. Also, the protocols in Slovenia for managing chronic patients need to be updated to include self-management support. Self-management support was slightly better implemented for T2D in Slovenia so more emphasis should be put also on HT patients. However, it is also important that community needs and opportunities are defined and that universal models are tailored to different settings.

This study has some limitations. The first one relates to the use of the tool to assess the implementation of the ICP. We adapted this tool from validated instruments [26,27], to be used for the specific conditions T2D and HT, but we did not validate our adapted tool as such. The tool was applied across the three countries, although minor adaptations were made to reflect local relevance. These limitations make the comparison of scores between countries and with studies in other countries difficult.

The second limitation is the purposive selection of units for ICP analysis within each country, so results should be interpreted with caution. The selection was made to capture variability within a country, but in more diverse health systems the results cannot be generalised for the entire country.

The third limitation could be the fact that we did not study the associations between quality of care and levels of integrated care implementation.

A fourth limitation is related to the evaluation of the ICP grid tool. The scores have been made by the national country team researchers, based upon their observations and interviews, which might be subject to observer and response bias. This was addressed through gathering data from different resources and using a method of consensus for ICP Grid scores.

Lastly, there are some considerations regarding the calculation of the scores. Namely, these were calculated differently among the three countries (in some countries, weighting was implemented) to use the methodology that was the most appropriate for each country. Also, our study was not aimed to directly compare the scores of the three countries, but to detect differences according to country’s health system and organisation of care.

Even if data collection span was quite large (extended to 2019–2022 due to COVID-19 pandemic) we believe that there was no time effect. Although integrated care implementation can evolve over time, during covid, the care was reorganised to mitigate the effect of the pandemic. One of the main activities that was disrupted or even stopped, was the chronic patient care [28]. Therefore, we believe that integrated care did not change in the years 2020 to 2022.

The way in which the assessment has been undertaken means that we have placed the ICP assessment tool in the context of the characteristics of the healthcare system. As the units analysed are not representative for each country, summative scores per country are not available for Belgium and Cambodia. The scores and the variation in scores give an indication of the differences in implementation within a country and allow us to reflect on the significance of the differences in scores between countries.

In designing future healthcare policies, several key considerations emerge from this study. Firstly, the importance of central policies and guidelines, as demonstrated in Slovenia [29], underscores the need for nationwide support for the implementation of integrated care packages (ICPs). This centralized approach provides a framework for consistency and standardization, yet it must be balanced with the flexibility to accommodate local contextual factors. Allowing decision-space for local adaptation enables healthcare organizations to tailor implementation strategies according to their specific needs and resources [30].

Resource constraints, particularly evident in settings like Cambodia [31], pose significant challenges to effective ICP implementation. Addressing shortages in competent staff and medication is essential to ensure the success of integrated care initiatives [32]. Furthermore, policies prioritizing quality improvement across diverse healthcare systems can enhance the implementation of ICPs, fostering better patient outcomes and healthcare delivery [33].

Standardized protocols and team compositions, as observed in family medicine practices in Slovenia [29], promote collaboration among healthcare providers. Encouraging such practices facilitates coordinated care delivery, which is essential for the effective management of chronic diseases [34].

Supporting health workers through training, resources, and practical aids is crucial for facilitating the implementation of ICPs and enhancing job satisfaction. Recognizing the pivotal role of nurses and community health workers in delivering health education [35] and self-management support emphasizes the importance of structured communication and tailored interventions.

Understanding the unique characteristics of each healthcare system and tailoring assessment methodologies accordingly allows for meaningful reflections on implementation variations within and across countries. By integrating these insights into future policy design, healthcare systems can better navigate the complexities of implementing and scaling up integrated care initiatives, ultimately improving patient care and outcomes.

## Conclusion

This paper uses a cross-contextual approach to assess the implementation of an ICP for two highly prevalent chronic diseases – T2D and HT. The variation of ICP implementation is not only large between different health system contexts, but also between different types of care models within the same health system. Differences in implementation relate to differences in health system resources and the organisation of the health system. National policies for health services support and local agility to steer resources in a way to meet local needs are both important facilitators for improved implementation and scale-up. Facilitators also include common guidelines well known to all providers in the country, multidisciplinary teams within one health care organisation and explicit programmes for involvement and empowerment of patients and sufficient and appropriately targeting financing. The analyses of context specific barriers to ICP implementation provide insights for improvements, both at the micro level of patients and providers, at the meso level of health care organisations and at the macro-level of health system arrangements.

Further studies should address the effect of integrated care on the quality of care of chronic patients.

## Implications for practice

Based on the results of this study, the countries can improve the implementation and scale-up of integrated care packages for chronic diseases, addressing contextual and health system factors to enhance patient outcomes and overall healthcare delivery. The interventions could include establishing central policies with guidelines for nationwide rollout, allowing local adaptation and decision-space, implementing standardized protocols and team compositions, addressing resource constraints by mobilizing local support, emphasizing systematic screening and health education, providing training and resources for healthcare workers, and tailoring support for self-management to community needs.
